# Addressing the 2050 demand for terrestrial animal source food

**DOI:** 10.1073/pnas.2319001121

**Published:** 2024-12-02

**Authors:** Alison L. Van Eenennaam

**Affiliations:** ^a^Department of Animal Science, University of California, Davis, CA 95616

**Keywords:** livestock, sustainability, cattle

## Abstract

The high emissions intensity of terrestrial animal source food (TASF) and projected increasing demand in low- and middle-income countries (LMIC) have spurred interest in the development of animal-free alternatives and manufactured food items that aim to substitute for meat, milk, and eggs with the promise of reduced environmental impact of producing food. The developing world is the source of 75% of global emissions from ruminants and will house 86% of the world’s human population by 2050. The adoption of cost-effective, genetic, feed and nutrition practices, and improving livestock health in LMIC are seen as the most promising interventions to reduce emissions resulting from projected increased TASF demand though 2050. Genetic improvement is a particularly attractive approach to productivity enhancements, as such improvements are permanent and cumulative. Alternative proteins may play a role in addressing demand for affordable sources of nutrient-dense foods, however, price will be a major factor influencing adoption given 3.1 billion people globally (42%) were currently unable to afford a healthy diet in 2021. Additionally, there is currently a mismatch between the location of alternative protein companies, and both projected increased TASF demand and emissions. To date, the vast majority (>81%) of these companies are based in high-income countries. The sustainability implications of replacing TASF with alternative proteins at scale needs to consider not only environmental metrics but also the wider economic and social sustainability impacts, given the essential role that livestock play in the livelihoods and food security of approximately 1.3 billion people.

Terrestrial animal-source foods (TASF) such as meat, milk, and eggs are nutrient dense and support the livelihoods and food security of almost 1.3 billion people, including almost 930 million Africans and South Asians (1). However, their production is associated with higher levels of greenhouse gas (GHG) emissions as compared to other foods (2, 3). A Food and Agriculture Organization of the United Nations (FAO) life cycle assessment (LCA) based on the reference year 2015 (4) found TASF were associated with emissions of 6.2 gigatonnes (Gt) of carbon dioxide equivalent (CO_2_eq), with over half being contributed by cattle (62%), followed by pigs (14%), chickens (9%), buffaloes (8%), and small ruminants (sheep and goats, 7%), equating to approximately 12% of all anthropogenic GHG emissions. High-income countries have developed efficient livestock production systems, which reduce both the cost and emissions intensity of animal products. However, these improvements have not been widely adopted in developing countries where the majority of livestock are located. It has been estimated that population and demand growth, primarily in Africa where the population is projected to increase by 80 percent by 2050 relative to 2020 levels, will drive a further 20% per capita increase in animal product demand by 2050. In the absence of any intervention or improvements in productivity, it is estimated that this increase will drive global livestock numbers and hence emissions to nearly 9.1 Gt CO_2_eq by 2050 (5).

One response to these projections could be to propose a rapid removal of animals from global food systems (6). While this would reduce GHG emissions, consideration of the needs and livelihoods of people in low- and middle-income (LMIC) countries are frequently absent from these discussions (7). In many LMIC households, diets are already predominantly plant-based because of the high price of nutrient-dense TASF. The cereal-based diets of the poor lack essential micronutrients, resulting in childhood stunting (low-height-for-age) which affected approximately 26.6% (21.5 to 32.4%) of children under five in 2017, particularly in sub-Saharan Africa and Asia, where stunting rates exceeding 30% still occur (8). Of the 176.1 million children who were stunted in 2017, over half lived in only four countries: India (51.5 million), Pakistan (10.7 million), Nigeria (11.8 million), and China (16.2 million) (9). Compared to plant-based foods, TASF supply higher quality protein and important micro- and macronutrients including vitamin A, vitamin B12, vitamin D3, iron, iodine, zinc, calcium, and folic acid (7). Rising incomes in LMIC are typically associated with a dietary shift from plant-sourced starchy staples to high-quality animal-sourced proteins from meat, eggs, and dairy, referred to the LMIC protein transition (10).

Previous work hypothetically modeled the nutritional and GHG implications of removing animals from US agriculture. It was found that while this did in fact decrease agricultural GHGs (28%) and total emissions (2.6%), plant-only diets formulated for the US population had an excess of dietary energy and resulted in a greater number of deficiencies in essential nutrients (11, 12). A less extreme approach that has been suggested by many authors is to decrease the intake of TASF, especially red meat consumption across wealthy and upper-middle-income economies (13), where typical diets include high amounts of TASF. This was modeled in the United States by replacing 10% of beef expenditures with other foods, or alternatively 10%, 30%, and 60% of beef expenditures were replaced with plant-based alternatives. This was found to reduce the number of animals needed for beef production by 2 to 12 million and reduced the carbon footprint of US food production by 2.5 to 13.5%. (14). This study did not examine nutritional implications of such a shift.

Behrens et al. (15) compared the dietary consumption patterns of average people with the nationally recommended food-based dietary guidelines (FBDG) in high-income and upper-middle-income nations and found FBDGs were associated with reductions in GHG of 13.0 to 24.8%, and 0.8 to 12.2%, respectively. The majority (54%) of the reduced environmental impact in high-income countries was driven by reductions in calories (i.e., eating the recommended number of calories rather than in excess of what is needed), and 46% by shifting to increased fruit, vegetables, and nuts at the expense of sugars, oils, meat, and dairy. Conversely, in LMIC, FBDGs were associated with increases in GHG by 12.4 to 17.0%. This was because of the recommendation for increased intake in TASF, partly due to the relatively high prevalence of undernutrition and micronutrient deficiencies in these regions (15). A systematic review of the literature about the relative health impacts of diets with reduced GHG emissions revealed that across all indicators of “healthiness”, 64% of lower GHG emission diets were linked to worse nutritional and health indicators (16). Reduced saturated fat and salt were often associated with diets low in animal products, but these diets were often also high in sugar and low in essential micronutrients.

When considering the timeline from 2020 to 2050, the estimated 20% increase in demand for TASF can be met by a proportionate rise in animal numbers, a stable or preferably decreasing number of more productive animals, alternative protein products, dietary changes, reduced waste, or more pragmatically some combination of all of these. In 2023, the FAO released a report (5) outlining the most promising interventions to cumulatively reduce projected GHG resulting from TASF demand by 55% though 2050, relative to a “business as usual” no mitigation scenario. These included improvements in animal and feed management, including productivity increases (20%), improved breeding (15%) and animal health (10%), adoption of known feed and nutrition practices (5%), and rumen manipulation with CH_4_ inhibitors (5%) (Fig. 1). This report also suggested that dietary shift had a limited reduction potential based on the fact that in LMIC, the typical diet often has low GHG because it falls below recommended calorie levels and lacks sufficient proteins, fruits, vegetables, and nuts. In those regions, a shift towards the recommended FBDG would be generally associated with increased overall consumption and a higher quantity of both plant- and animal-based foods (12). Given that population growth, and the need for increased dietary levels of TASF is occurring in LMIC countries, and that these countries are home to 76% of the global cattle herd and contribute 75% of the global ruminant GHG emissions (17), this would seem to be the logical place to focus emission reduction efforts. Increasing livestock productivity generally requires simultaneous interventions in the areas of animal feed, health, and genetics (18).

**Fig. 1.**
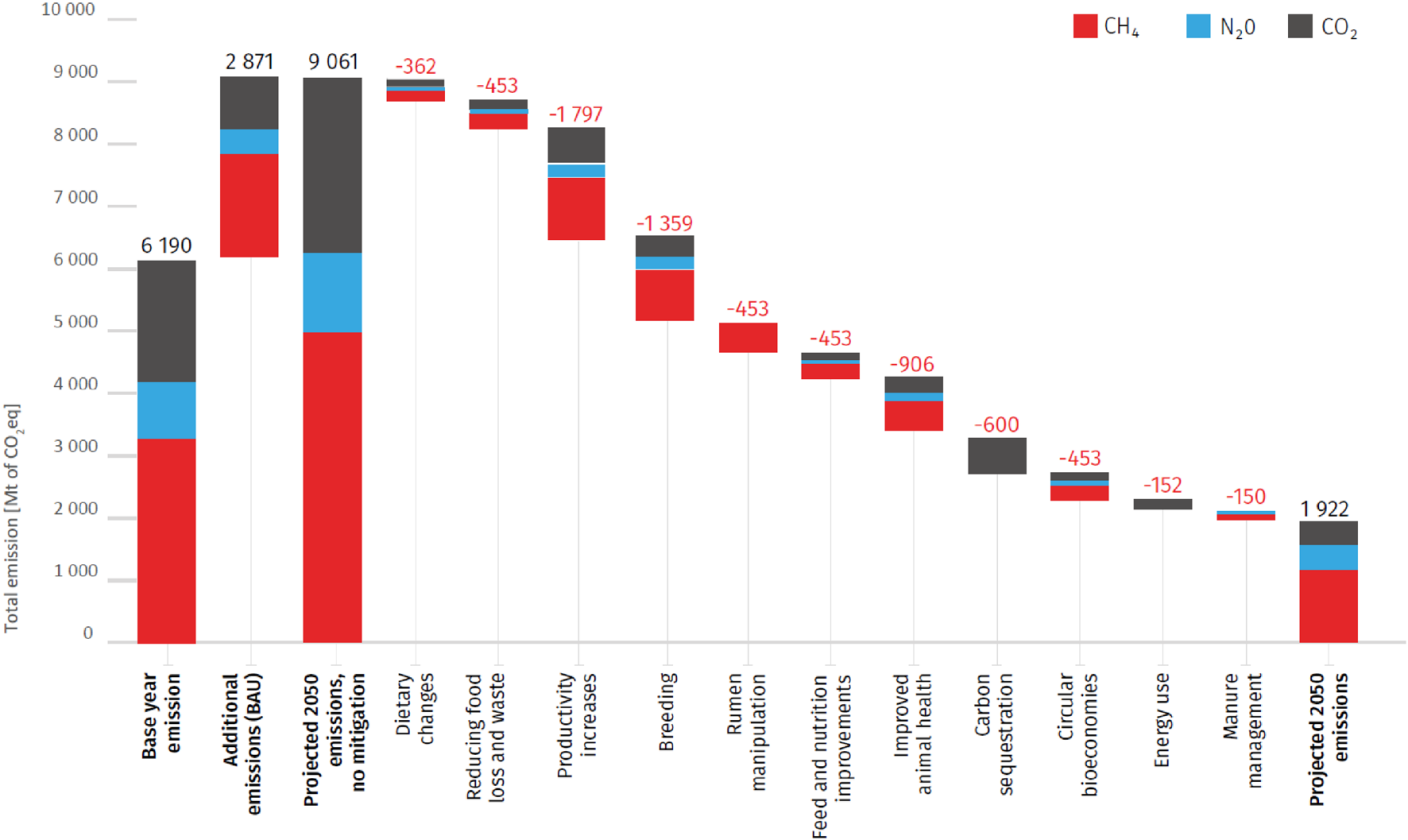
Base year and projected emissions from livestock systems shown as a waterfall chart with a range of mitigation measures applied to 2050 with their technical potential. Image comes from FAO. 2023. Pathways towards lower emissions – A global assessment of the GHG emissions and mitigation options from livestock agrifood systems. Rome https://doi.org/10.4060/cc9029en and is published under the CC BY-NC-SA 3.0 IGO license.

In 2021, the global cattle (152.9 billion) and buffalo (203.9 million) populations collectively produced 74.9 Mt meat and 757 and 149 Mt of milk, respectively (19). The majority (59%) of beef is produced in mixed crop and livestock systems, as compared to only 7% (2% global cattle population) in intensive feedlot systems, with the remaining 34% being produced in grazing systems (20). This latter group can be further divided, first into intensive grazing systems that are found in tropical and temperate zones where high-quality grasslands and fodder production can support larger numbers of highly productive animals (e.g., Australia). These systems are mostly focused on food production, based on individual landownership, and supply about 20% of global beef production. The second category are pastoral livestock grazing systems that have developed in harsh environments, such as dry lands and cold areas, and which account for less than 15% of total beef production, but which support the livelihoods of 200 to 500 million pastoralists (21). Mixed crop–livestock systems are responsible for 58% of total emissions, whereas grazing-based systems contribute 19% (22). Industrial and other systems comprise the remaining 23%.

The intake of ruminants in grazing and mixed systems is mainly composed (about 90%) of non-human edible roughage: leaves, grass, silage, and crop residues (23). Ruminants need 0.6 kg of human-edible protein feed intake to produce 1 kg of protein (as compared to the 2 kg requirement of monogastrics), which makes them net contributors to global human-edible protein production. The FAO estimates permanent meadows and pastures comprise 67% of the 4.8 billion hectares that are used for agriculture globally. Of the 3.5 billion hectares of permanent grasslands globally, 1.5 billion are not suitable for grazing, and 2 billion hectares are grazed by ruminants. Of these, 1.3 billion hectares of pastures and rangelands are considered marginal, meaning they are not suitable to be converted to croplands or forests due to climatic, soil fertility, and topographical factors (23). Ruminants are uniquely positioned to convert this cellulosic biomass into nutrient-dense TASF. This is part of ruminants’ role in circular food systems (24). In their absence, this land would produce no human food.

Where and how cattle are raised has important implications on the emissions intensity of production. Innovations in the three main disciplines of animal science, genetics, nutrition, and health, historically supported by extension programs that facilitate adoption of best practices, have been associated both with efficiency gains and large decreases in the emissions intensities (emissions produced per unit of product) in high-income countries (25). However, this improvement has not been universal, and many LMIC have production systems with high emissions intensities (26). The oft-cited global average of 99.5 kg CO_2_eq/kg beef (3) hides considerable regional variation. Emissions intensity can vary 50-fold among producers of the same product in similar geographic regions. Chang et al. (27) estimated that improving livestock production efficiencies in the 10 countries with the largest emission reduction potential (Madagascar, Morocco, Niger, South Africa, Tanzania), Asia (China, India, Iran, Turkey), and South America (Brazil) could contribute 60% to 65% of the global reduction in livestock emissions by 2050 (compared to a baseline where emissions intensities are held constant in the future). These authors reported that efforts to improve production efficiencies would have a much greater potential for GHG mitigation than demand-side efforts to promote balanced, healthy, and environmentally sustainable diets. These efforts alone would not be sufficient to mitigate TASF emissions.

It is worth noting that the production of beef is not proportional to the size of the cattle population. The United States produced 18% (12.7 Mt) of the world’s beef with 6% (93.8 million) of the world’s cattle in 2021. Brazil produced 13% (9.75 Mt) of the word’s beef with 15% (225 million) of the world’s cattle population, whereas India with 193 million cattle and 112 million buffalo produced only 5.8% (4.20 Mt) of bovid meat. Of particular importance is the African continent population, which produced around 9.5% of the world’s beef with almost 25% (373 million) of the world’s cattle (19). There are a myriad of reasons why beef production is not the main driver of cattle numbers, including the fact cattle are also raised for milk and other coproducts like hides.

Most carbon footprint assessment studies focus only on edible products and do not account for the multifunctionality of livestock. In many regions, particularly in LMIC, livestock are not only raised for food production but also for their role in providing draft power, serving as a financial asset and as a means of saving. Additionally, cattle in India have religious significance which forbids the consumption of beef, and in Africa, manure production and animal traction are key functions served by cattle for crop production in mixed systems (28). In other words, cattle are not raised solely, or even primarily, for beef in many countries, and hence, beef production efficiency is not the main objective of these farmers. Evaluating these systems based on metrics that value edible products alone ignores the multifaceted roles that ruminants play in global agri-food systems. Some have suggested that GHG emissions in such systems should be allocated across the multiple functions to appropriately acknowledge nonsalable products and other roles that cattle play in smallholder systems (29). These authors argue that using a traditional single-issue LCA (i.e., kg CO_2_eq) in “complex mixed smallholder dairy systems with multifunctional aspects related to cattle keeping” does not “grasp the system for what it is. It follows that policy recommendations stemming from such a fundamental misapprehension of smallholder agricultural practice will be misguided, at best”, and that a livelihoods lens is needed to define “productivity” (29).

Moreover, beef is actually not even half of the picture with respect to cattle numbers, because milk constitutes 67% of the total protein produced by cattle. India produces 24% (214 Mt) of the world’s buffalo and cattle milk, followed by the United States 11.3% (103 MT), Pakistan 6.5% (59 Mt), China 4.4% (40 Mt), and Brazil 3.9% (36 Mt). About half of India’s milk production is from water buffalo, and the other half is from cattle. The European Union as a collective produces around 25.1% (227 Mt) of the world’s milk with 5% (76 million) of the world’s cattle population and Africa as a collective 4.7% (42 Mt) of milk (19). World milk production is projected to grow at 1.5% p.a. over the next decade to 1 085 Mt in 2033 (30), faster than most other main agricultural commodities. Over half of the increase in total milk production is anticipated to come from India and Pakistan, which will jointly account for over 32% of world production by 2032. According to an OECD report (30), “the global level of GHG emissions will largely depend on efficiency gains in India and other countries with high cattle populations and extensive production.”

## Opportunities in Breeding and Genetic Improvement

The FAO report (5) detailed a number of promising interventions (improved livestock diets, genetics, veterinary care, and management practices) to reduce projected GHG resulting from TASF demand by 55% though 2050. It should be noted that this report assumes a full adoption of intervention, illustrating what is possible (technical mitigation potential), rather than the extent to which these practices will be adopted (economic mitigation potential) (25). As a geneticist, I am going to focus on opportunities in breeding as a particularly attractive approach to productivity enhancements, as following implementation genetic improvements are permanent and cumulative (31). There are some relatively simple technologies, such as altering the sex ratio of offspring, and using artificial insemination (AI) to increase the use of genetically superior bulls (32), that can have major impacts on the efficiency and carbon footprint of cattle production systems. First introduced in the early 1940s, AI has been widely adopted in the dairy industry of many high-income countries, helping to achieve a more than two-fold decrease in the GHG per kg of milk in the United States from 3.66 in 1944 to 1.35 in 2007 (33). However, the adoption of AI has not been universal and is particularly low in LMIC and extensive beef production systems where it is difficult to coordinate estrus synchronization protocols and perform AI (34). The following section details efforts to increase AI adoption in Brazil and India, the two individual countries with the largest global cattle populations. Additionally, in high-income countries, the recent adoption of genomic testing and sexed semen has resulted in a dramatic change in the origin of calves entering the beef supply chain (35), with further sustainability implications for cattle production in those countries.

## Lessons from India and Brazil

By 2050, India’s human population is projected to reach 1.65 billion which represents a 16% increase from the current figure of 1.43 billion people, of which 8.8% currently rely on the livestock sector for their employment, and 20.5 million individuals for their livelihoods (36). Milk yield per cow was only 424 kg/y in 1961, limited by a shortage of quality feeds and fodders and few high-yielding milk cows. However, yield increased more than four-fold to 1777 kg per animal in 2019–2020, still shy of the global average of 2,699 kg. Part of this was due to substantial government investment, in partnership with the World Bank, in Operation Flood from 1970 to 1996. In 30 y, it transformed India from a milk-deficient nation into the largest milk-producing country in the world. This growth in the dairy sector was both pro-poor and pro-women, having multiple beneficial effects including nutrition, education (especially of girls), and job-creation, and is helping India to make progress toward sustainable development goals (SDGs) addressing poverty and promoting economic growth (36). Milk consumption by children aged 6 to 59 mo in India, a country with 24% of undernourished children worldwide, has been associated with reduced odds of stunting, underweight, and anthropometric failure (37). The World bank calculated that for an initial investment of 2 billion rupees in Operation Flood II, the net return/year to rural economy was 240 billion rupees (38). This is a very high input–output ratio for a development program. According to the World Bank, this was due in part to the fact that this project “encouraged members to invest in biological assets—milk cows—that periodically reproduce themselves without major reinvestment and continue to yield regular benefits, utilizing crop residues that otherwise do not have much economic value” (39).

**Fig. 2.**
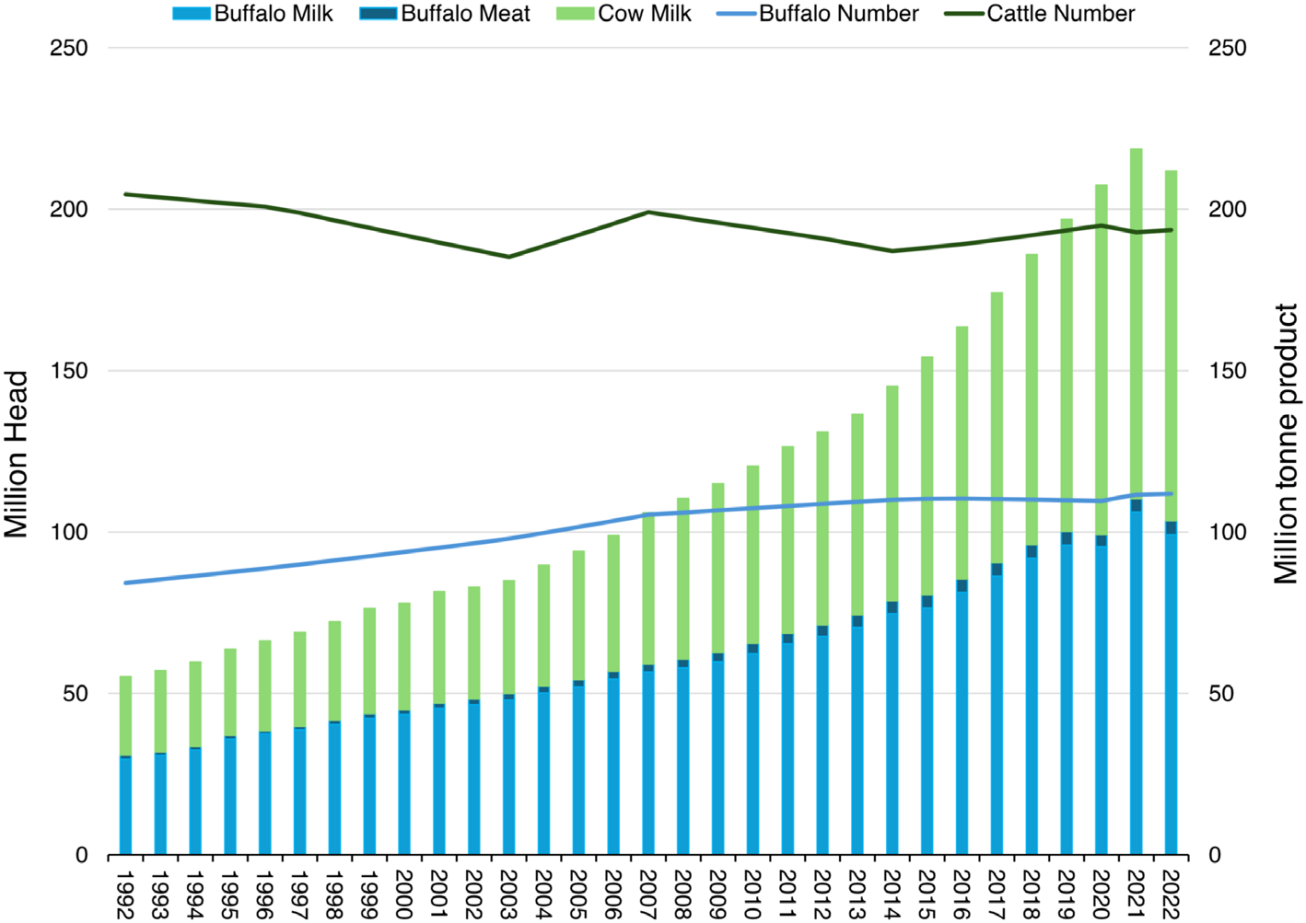
Cattle and buffalo numbers and production of meat and milk 1992-2022 in India. FAO 2022. FAO Stat: Crops and livestock products. Accessed 15/10/2024. https://www.fao.org/faostat. License: CC-BY-4.0.

This increased milk yield per cow was partly a result of improved genetics enabled by the use of AI starting in 1985 and which continues to be the backbone of bovid breeding programs in India (40). The total number of AI performed in India increased five-fold this century from 19.77 million in 2000–2001 to 98 million in 2021–2022. Even so, the overall AI coverage rate in bovines in India remains relatively low at ~30% with a conception rate of 35% (40), and AI coverage rates on buffalo in India and Pakistan are 35% and 14%, respectively, suggesting considerable room for improvement given the right production incentives, investment, and institutional support. The percentage of crossbred dairy cows rose from 17% in 1990–91 to 38.3% in 2021–22, with their contribution to total cow milk production increasing from 33.5% to 61.2% (41). Concurrently, there has been a noticeable trend toward raising more female cattle. The number of female cows climbed from 102.98 million in 1992 to 145.12 million in 2017, while the number of male cattle fell from 101.59 million to 47.4 million. Since 1997, the country's milk production has increased an additional 140 Mt (306%), driven mostly by improved productivity per cow in combination with a comparatively modest 6% increase (~ 16.67 million head) in the overall bovid population, characterized by a decline of 5.28 million cattle and an increase of 21.9 million buffalo (Fig. 2). The use of X chromosome sorted “sexed” semen in combination with AI so as to increase the likelihood of producing a female calf (36) is a particularly interesting proposition for the India dairy sector (41).

Brazil has the largest cattle population in the world at approximately 225 million head, composed of 43% dairy and 57% beef cattle. Interestingly, AI is more frequently used in the beef industry than the dairy industry in this country. Uptake of AI in the Brazilian beef industry has been very much driven by the development of protocols for timed artificial insemination (TAI) which allows a scheduled insemination of animals without the need to detect estrus. Using TAI facilitates increased both pregnancy rates early in the breeding season and the genetic merit of beef calves, and in 2023 91.2% of inseminations in Brazil were performed using TAI (42). The return on investment of TAI technology in the beef and dairy production chain in Brazil is 4.5:1. Despite the economic benefits of the usage of TAI programs, many farms do not use it as they do not have adequate animal handling facilities or specialized personnel to implement it (43). The increased use of TAI and concomitant genetic improvement in the Brazilian beef industry can be seen to coincide with technological modernization and improved production practices including integrated production systems, new forages, management and recovery of pastures, and feed supplementation. From 1990 to 2017, Brazil went from producing 24.45 kg of live weight/ha/y to 60.15 kg of live weight/ha/y (44).

Despite the fact that AI is a technology that clearly boosts livestock productivity and generates income, adoption rates in LMIC remain low. Adoption depends on the costs and logistics of implementation, in addition to public and private incentives, policies, and taxes. Sexed semen adoption in developing countries is currently low due to its lower conception rate ~ 10 to 15% relative to conventional semen, and the high cost (~ three to five times more) as compared to conventional semen (45). Both the Indian and Brazilian governments have made significant investments in programs to increase the use of AI. The Indian government has launched a 5-y program 2021–2026 which aims to bring breeding services to perform AI on 165 million animals, train AI technicians, produce high genetic merit bulls, increase the production of bovine semen, and subsidize 50% of the cost of sorted semen with the goal of establishing 510,000 pregnancies, of which 90% would be expected to be female. In 2002, the Brazilian market for AI was approximately 7.0 million doses of semen. 20 y later (2023), 24.7 million doses of semen were commercialized, and 20 to 23% of the females in the national herd were inseminated. It is estimated that the percentage of inseminated cows will increase to 37% in the next 10 y (42).

## The Generation of Crossbred Beef × Dairy Cattle

In high-income countries, the rapid adoption of two new technologies in the past decade has resulted in a dramatic change in the type of beef produced in dairy production systems, with further sustainability implications for the environmental footprint of beef production (35). The combination of genomic selection to enable identification of genetically superior commercial dairy cows, which has doubled the rate of genetic improvement in the US dairy herd in the past decade (46), and X-sorted sexed semen has allowed dairy producers to target the development of replacement heifer offspring to their genetically superior cows, typically young heifers. That has opened up the possibility of using embryos or semen from beef cattle breeds to impregnate low genetic merit dairy cows to produce calves that are better suited to beef production than the male and surplus female dairy calves that were historically destined for veal, pet meat, or even on-farm euthanasia. On average, beef produced in specialized beef herds has four times the emissions intensity of so-called “dairy beef” which has historically been a by-product of dairy production, comprised mainly of old cows at the end of their productive life (47). Most of the emissions from specialized beef herds come from the breeding stock (i.e., cows) that produce no product other than weaning a calf.

A simulation study investigated the sustainability implications of generating more beef calves from the dairy sector in Ireland. In this scenario, it was found that the widespread usage of sexed semen (fertility equal to conventional) on the genetically top third of dairy cows and heifers to produce female replacements, combined with the use of beef semen used on the bottom two-thirds could reduce the Irish carbon footprint of beef by 24.6%, from 17.4 to 13.1 kg CO_2_ eq/kg carcass weight by 2030 (48). Additionally, it was found that increasing the beef derived from the dairy herd from 50% to 75% would lead to a 23% reduction in GHG emissions. There are other advanced breeding innovations such as the selection for low methane-producing ruminants (49), genome editing for adaptability traits like disease resistance (50) and heat tolerance (51), and even technologies like surrogate sires (52) to promote the distribution of elite genetics via natural service mating in extensive pastoral industries that could further help further reduce the emissions intensity of beef and milk.

The majority of genetic improvement strategies have been designed in developed countries and rely on the foundation of a well-structured breeding program that captures animal performance phenotypes and matches them with a database of genotypes. There are few such recording schemes in LMIC due to small herds, incomplete recordings for most traits, no parentage recording, and insufficient contemporary groups, although there are efforts to develop them (53, 54). The importance of TASF in global food systems is not always well-reflected in development spending. Less than 20% of agricultural research and development expenditures from 1992 to 2016 by the Consultative Group for International Agricultural Research were directed to livestock and fisheries, with most being directed toward research to improve yields of staple crops (55). Targeted investments aimed at boosting animal productivity—such as advancements in technology and the adaptation of livestock genetics, feeds, and health solutions—can play a crucial role in development strategies that promote human welfare and improve natural resource management (56). There are very few studies that look at the triple bottom line of social, economic, and environmental consequences of adopting practices that reduce GHG emissions intensities in livestock systems (57), especially in LMIC. The intensification that typically occurs to reduce emissions intensity through increased feed availability and feeding practices and genetic improvement (58) are associated with social and economic trade-offs as well as synergies, and these should also be taken into account to avoid maladaptive outcomes.

## Alternative Proteins

Alternative proteins (proteins from algae, plant-based products, mycoproteins, insects), fermentation-based proteins, and animal cell-based proteins have been proposed as a more sustainable approach to produce food that can replace, substitute for, or augment TASF production (59). The sustainability framing often involves a narrative using a metric like CO_2_eq GWP_100_ per unit weight of product to compare alternative products to TASF values (60). More generally, the literature on sustainable diets predominately uses GHG as a proxy for sustainability and focuses mostly on high-income countries (61), overlooking the production and dietary alternatives most relevant to LMIC. Some consider that alternative proteins will be a niche market that complements or augments TASF (62), whereas others project that alternative animal products will help feed the world and significantly diminish the effects of climate change (63). One CEO confidently pronounced on a global stage that the goal of his alternative protein company was “to completely replace animals in the food system by 2035” and promised plant-based substitutes “for every animal product used today in every region of the world” (64). Given the importance of livestock in global food systems, such claims from a single US-based company should rightfully be challenged by scientists with expertise and training in the agricultural sciences. For context, global plant-based meat alternative sales reached $5.6 billion in 2021, whereas global conventional meat markets were valued at $838.8 billion in 2020. Global cultured meat sales are predicted to begin following approvals in US and Singapore and reach $352.4 million by 2028, with North America expected to have a 35% market share (65). Global meat and dairy production was 354 Mt and 927 Mt in 2023 and is projected to rise to an estimated 388 Mt (110%) and 1 085 Mt (117%) by 2033, respectively (30). There are few peer-reviewed reports of the predicted size of the future alternative proteins market, but one prediction estimated it to grow from US $17.97 billion in 2024 to US$ 50.45 billion by 2035 (https://www.rootsanalysis.com/alternative-proteins-market; Accessed 10/17/2024).

The NAS consensus study report, “Science Breakthroughs to Advance Food and Agricultural Research by 2030” (66) stated that “There is a need to objectively evaluate the sustainability implications of different animal agricultural systems and protein source alternatives using a holistic evidence-based research approach”. Multiple scientists have written on the trade-offs that would result from dramatically reducing or eliminating global animal populations, or removing ruminants from lands unsuited to crops, and the need for a multidisciplinary, balanced examination of the implications of such a shift on many of the UN’s SDG (7, 23, 24, 67–73). However, the multitude of complex trade-offs that should legitimately be part of scholarly examinations around differing food production systems and dietary shifts are often simplified down to GHG emissions per unit weight of product, with no consideration given to the nutrient composition or quality of either the inputs or the outputs. The perspectives and knowledge brought by experts in animal agriculture to global food system discussions are frequently dismissed (69, 74). Prominent voices in media, policy, and academia have amplified the claim that there is a settled scientific consensus that animal agriculture is a global problem that needs to be downsized, rather than optimized. In protest to this contention, over 1,200 researchers signed the Dublin Declaration on the Societal Role of Livestock (75). The purpose of this effort was “to give voice to the many scientists around the world who research diligently, honestly and successfully in the various disciplines in order to achieve a balanced view of the future of animal agriculture” (75).

**Fig. 3.**
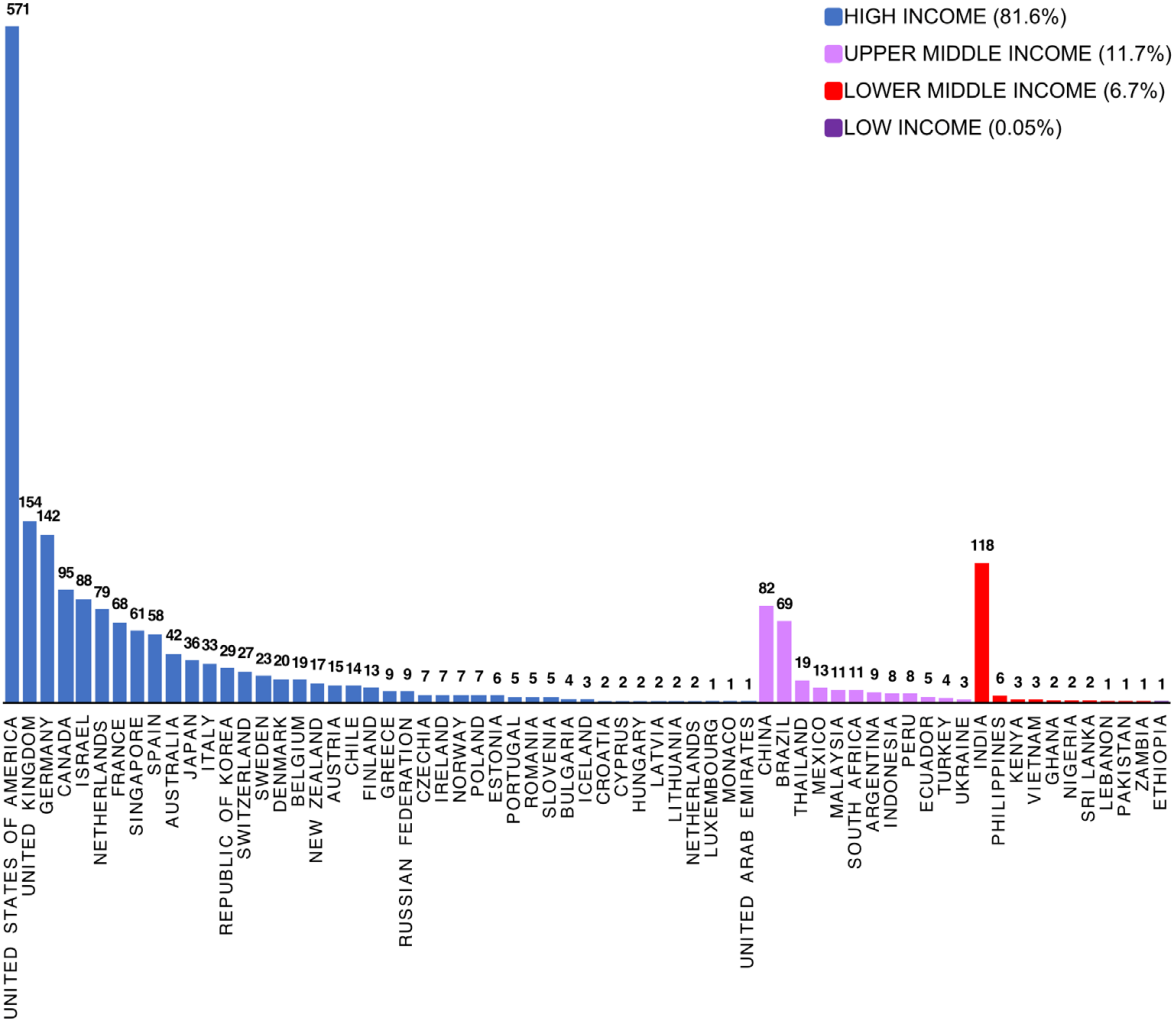
Global Distribution of Alternative Protein Companies (n = 2,075) by World Bank Country Income Level Classification. Based on alternative protein company database containing 2,099 records, maintained by The GFI https://gfi.org/resource/alternative-protein-company-database/ (Accessed 10/17/2024).

If alternative proteins are being proposed to replace TASF, this transition should be assessed using an approach that captures the triple bottom line of social, economic, and environmental consequences, particularly in LMIC. Only 16% of the world population currently lives in high-income countries, and the share of people living in developing countries is expected to increase to 86% (8 billion people) by 2050 (76). Increased demand for TASF is predominately occurring in LMIC countries. However, according to an alternative protein company database containing 2075 unique records, maintained by The Good Food Institute (GFI) (https://gfi.org/resource/alternative-protein-company-database; Accessed 10/16/2024), 82% of these companies were based in high-income countries, 12% in upper middle-income countries, 7% were in lower middle-income counties, and one (0.05%) was in a low-income country (Fig. 3). At the beginning of 2022, there were about 112 companies worldwide involved in “cultured meat” production. The location of these companies include 20 in the EU, 14 in the United Kingdom, 33 in North America, 22 in Asia (9 in Singapore and 4 in China), and 14 in Israel (62).

Additionally, if alternative proteins are going to supplement or replace some TASF at scale, considerable capital will be required to construct manufacturing facilities. In 2021, the GFI with support from companies active in the field estimated that a commercial-scale facility to produce 10,000 Mt of ground-cultivated meat product per year would cost US $450 M to build (77). Another estimate puts this number at US $600 M (78). A recent study modeled a theoretical beef production facility producing 100,000 Mt of cultured beef and calculated the capital expenditure to be between $1.5 billion and $10.95 billion depending upon reactor size (79). It has been estimated that for global consumption of alternative proteins to reach a baseline of 97 million Mt of product by 2035, it would require up to $11 billion to fund the extrusion capacity needed for plant-based proteins and up to $30 billion in investment capital for the bioreactor capacity needed for 30 Mt of microorganisms and animal cells (80). The GFI estimates that over the past decade, the alternative proteins sector attracted U$16 billion globally in private capital (https://gfi.org/investment/; Accessed 6/15/2024).

Moreover, unlike animals that “can periodically reproduce themselves without major investment” (39), factory and infrastructure depreciation costs will also have to be factored into the cost of producing alternative products. A techno-economic modeling study estimated depreciation costs between $1 and $10/kg of the cultured meat costs of goods, depending upon the capital costs of the factory (79). This cost alone would be problematic for much of the world. It was estimated in 2021 that for over 1.5 billion people, the $2.42 median cost of the EAT-lancet reference diet in low-income countries would exceed their total daily income (81). More generally, over 3.1 billion people (42%) were unable to afford a healthy diet in 2021 (82). If alternative proteins are intended to contribute to food security in LMIC and “promise both nutritional salvation and economic development for the hungry poor” (60), there is currently a mismatch between the location of these companies and projected TASF demand. The role of alternative proteins to address the LMIC protein transition is likely to be limited due to availability, costs, and cultural preferences. Improved livestock farming systems are seen as a more promising approach to address this demand, while creating on-farm opportunities for income growth and jobs, improving both food security and fostering economic development in LMIC (28).

## Conclusion

The discussion around TASF is frequently focused on a narrow subset of environmental metrics and first world consumption patterns, with little regard for locations where livestock underpin the necessities of life. There are a myriad of functions that animals provide in global food systems in addition to the provision of TASF, and these include supporting crop production with draft power and manure, providing a valuable use for crop residues and other by-products, generation of a regular income and employment especially for women, the provision of food security insurance and a form of savings, as well as fulfilling cultural and social roles. Efforts to promote sustainable diets need to be assessed using an approach that captures the triple bottom line of social, economic, and environmental consequences. This is especially important in LMIC, given that these regions are the projected epicenter of human and livestock population growth, and concomitant increases in both TASF demand and livestock-related GHG emissions through 2050. Improvements in animal genetics, health, and feed management are seen by a recent FAO report to be the most promising interventions to cumulatively reduce projected GHG resulting from TASF demand though 2050.

## Data Availability

There are no data underlying this work.
